# Development of a Cost-effective Ovine Polyclonal Antibody-Based Product, EBOTAb, to Treat Ebola Virus Infection

**DOI:** 10.1093/infdis/jiv565

**Published:** 2015-12-28

**Authors:** Stuart David Dowall, Jo Callan, Antra Zeltina, Ibrahim Al-Abdulla, Thomas Strecker, Sarah K. Fehling, Verena Krähling, Andrew Bosworth, Emma Rayner, Irene Taylor, Sue Charlton, John Landon, Ian Cameron, Roger Hewson, Abdulsalami Nasidi, Thomas A. Bowden, Miles W. Carroll

**Affiliations:** 1 Public Health England, Salisbury; 2 MicroPharm,Newcastle Emlyn; 3 Division of Structural Biology, Wellcome Trust Centre for Human Genetics, University of Oxford,United Kingdom; 4 Institute of Virology, Philipps University Marburg, Germany; 5 Nigerian Centre for Disease Control, Abuja

**Keywords:** Ebola, therapeutic, neutralization, antibody, efficacy

## Abstract

The highly glycosylated glycoprotein spike of Ebola virus (EBOV-GP_1,2_) is the primary target of the humoral host response. Recombinant EBOV-GP ectodomain (EBOV-GP_1,2ecto_) expressed in mammalian cells was used to immunize sheep and elicited a robust immune response and produced high titers of high avidity polyclonal antibodies. Investigation of the neutralizing activity of the ovine antisera in vitro revealed that it neutralized EBOV. A pool of intact ovine immunoglobulin G, herein termed EBOTAb, was prepared from the antisera and used for an in vivo guinea pig study. When EBOTAb was delivered 6 hours after challenge, all animals survived without experiencing fever or other clinical manifestations. In a second series of guinea pig studies, the administration of EBOTAb dosing was delayed for 48 or 72 hours after challenge, resulting in 100% and 75% survival, respectively. These studies illustrate the usefulness of EBOTAb in protecting against EBOV-induced disease.

Ebola virus (EBOV) is the prototype species of the genus *Ebolavirus* in the Filoviridae family [1, 2] and responsible for the large outbreak of EBOV disease in parts of West Africa, since being recognized in March 2014 [3]. The unprecedented number of mortalities associated with this outbreak emphasizes the need for improved therapeutic measures.

Several recent studies have focused on the therapeutic development of monoclonal antibodies (mAb) [4], including ZMapp, a cocktail of 3 chimeric mAb that target distinct epitopes on the EBOV glycoprotein (GP_1,2_) surface [5]. The use of human (homologous) polyclonal antibodies (pAb) from convalescent patients has also shown promise in the treatment of EBOV infection [6, 7] and is the first form of immunotherapy for EBOV approved by the World Health Organization [8]. Human-derived mAb or pAb have the advantages in that they do not usually induce hypersensitivity or other side effects and have a long circulating serum half-life. Additionally, mAb cocktails and pAb target multiple nonrelated epitopes, thereby diminishing the risk of intrahost antigenic variation on the EBOV-GP_1,2_ surface [9, 10] that may impede their efficiency. However, human-derived antibody treatments suffer from issues with scalability, testing for the presence of other pathogens, and operating within difficult environments that lack equipment infrastructure and trained personnel [11]. Therefore, an alternative approach is necessary.

Heterologous (animal-derived) pAb have been used successfully for over a century to treat a range of conditions, including rabies [12] and tetanus [13]. However, there is a paucity of studies relating to their use in EBOV infections. Recently, Chippaux et al [14] proposed a revival of using heterologous pAb, noting the successful use of such reagents in Africa for therapeutic antivenoms. Importantly, in addition to being highly effective, pAb can be produced rapidly and affordably, constituting an economically viable option for developing regions facing epidemic EBOV disease. For >15 years, with initial support from the Nigerian Federal Ministry of Health, MicroPharm supplied an intact ovine immunoglobulin G (IgG)–based antivenom EchiTAb, which has been used to treat >40 000 patients envenomated by *Echis ocellatus* in West Africa. As such, EchiTAb is one of the most cost-effective therapies currently available [15]. Thus, it was appropriate to develop an intact ovine IgG–based product for the treatment of EBOV infections.

## MATERIALS AND METHODS

### EBOV-GP_1,2_ Expression and Purification

The complementary DNA (cDNA) of the GP from the EBOV Mayinga variant (GenBank accession no. U23187.1) was produced synthetically (GeneArt, Regensburg, Germany), and a construct corresponding to the EBOV GP ectodomain (residues M1-D632) was cloned into the pHLsec mammalian expression vector [16]. For protein expression, human embryonic kidney (HEK) 293 T cells were transiently transfected in roller bottles with 2 mg of purified EBOV-GP_1,2ecto_ DNA per 1 L of 90% confluent cells by using polyethyleneimine (PEI), with a DNA to PEI mass ratio of 1:2.

Cell supernatant was harvested 4–5 days following transfection. Cell debris was clarified, sterilely filtered through a 0.22-µM membrane filter, and diafiltrated against a buffer containing 10 mM Tris (pH 8.0) and 150 mM NaCl. EBOV-GP_1,2ecto_ was purified by immobilized metal affinity chromatography (IMAC), using Chelating Sepharose Fast Flow Ni2+-agarose columns (GE Healthcare, Buckinghamshire, United Kingdom) and desalted using a HiPrep 26/10 Desalting Column (GE Healthcare) against a buffer containing 10 mM Tris (pH 8.0) and 150 mM NaCl, concentrated, and sterilely filtered for immunization. For Western blot analysis, proteins were detected with mouse PentaHis antibody (Qiagen, Crawley, United Kingdom) and visualized by chemiluminescence of a secondary anti-mouse horseradish peroxidase antibody (Sigma Aldrich, Manchester, United Kingdom).

### Antisera Production

The immunogen for the primary immunization comprised Freund's complete adjuvant and 500 µg of EBOV-GP_1,2ecto_ per sheep. The protein:adjuvant mixture was injected subcutaneously and equally into 6 injection sites chosen for their proximity to the axillary, inguinal, and prescapular drainage lymph glands. Each sheep was reimmunized at 28-day intervals with 500 µg of EBOV-GP_1,2ecto_ and Freund's incomplete adjuvant, and blood samples were collected 14 days later. A total of 10 mL of blood per kg of body weight can be collected from the external jugular vein without detriment to the animal.

### Manufacture of EBOTAb

The IgG fraction was purified from the ovine antisera by adding caprylic acid (octanoic acid) at a concentration of 6% v/v and then further diluting with 2.0 parts saline to precipitate non-IgG proteins [17]. The product was formulated at a concentration of 50 g/L in 20 mM citrate buffer, containing 153 mM NaCl (pH 6.0 ± 0.2). It contained no preservatives or stabilizers. Purity was assessed by size-exclusion gel filtration chromatography, using a Pharmacia AKTA/FPLC chromatography system with a Pharmacia Superose 12 HR 10/30 column, equilibrated and eluted with 20 mM sodium citrate saline (pH 6.0) containing 153 mM NaCl, at a flow rate of 0.5 mL/minute [17].

### Enzyme-Linked Immunosorbent Assay

Immulon 4HBx microtiter plates were coated with 2 µg/mL EBOV-GP_1,2ecto_. Plates were washed with 3 changes of phosphate-buffered saline (PBS) containing 0.1% Tween 20 and 0.01% thimerosal (PBST) and blocked for 1 hour at 37°C with blocking buffer (2.5% fetal calf serum prepared in PBS). Plates were washed and incubated for 1 hour at 37°C with antisera at initial dilutions of 1:1000, followed by 1:2 serial dilutions; washed with PBST; and incubated with a donkey anti-ovine IgG horseradish peroxidase conjugate for 1 hour at 37°C. After further washing, TMB substrate solution was added, and the reaction was stopped after approximately 10 minutes by the addition of 1.0 M HCL before reading the optical density at 492 nm.

### Small-scale Affinity Chromatography (SSAC)

SSAC was used to determine the specific antibody content [18]. Briefly, 5 mg of EBOV-GP_1,2ecto_ prepared in 5 mL of coupling buffer (0.1 M sodium carbonate [pH 8.3]) was coupled to cyanogen bromide–activated 4 Fast Flow Sepharose (GE Healthcare). After draining the uncoupled protein, the remaining active groups were blocked with 1 M ethanolamine and packed onto a small glass column (BioRad, United Kingdom) and washed with assay buffer (10 mM sodium phosphate containing 0.5 M NaCl and 15 mM sodium azide [pH 7.4]) followed by elution buffer (0.1 M glycine [pH 1.5] containing 0.1 M HCl) and stored at 4°C until further use. One milliliter of ovine antisera or EBOTAb was added to the column and mixed end over end for 2 hours at ambient temperature. The column was subsequently washed with assay buffer and eluted, and the specific antibody content was determined spectrophotometrically at 280 nm (1-cm path length), using an extinction coefficient of 1.5 for ovine IgG.

### Western Blotting

Lysates from EBOV-infected or uninfected VeroE6 cells were subjected to sodium dodecyl sulfate polyacrylamide gel electrophoresis (SDS-PAGE) on a 4%–12% Bis-Tris gel (Life Technologies) and transferred to a polyvinylidene difluoride membrane. After blocking in 5% milk protein, membranes were incubated with EBOTAb for 2 hours, washed 6 times with PBS containing 0.05% NP40, incubated for 1 hour with horseradish peroxidase–conjugated rabbit anti-ovine IgG (Sigma), and washed as before. All antibody dilutions were made in PBS containing 0.05% NP40 and 5% milk protein. Bound antibody was detected with ECL-Prime WB detection reagent (GE Life Sciences, United Kingdom) according to the manufacturer's directions and visualized on a ChemiDoc system (BioRad). Molecular weights were calculated by comparison with markers of known molecular weight.

### Neutralization Assay

EBOTAb (51.5 mg/mL) or a nonspecific control antibody (26 mg/mL) were serially diluted from 1/2^3^ to 1/2^14^ in 96-well culture plates. One hundred 50% tissue culture infective doses (TCID_50_) of EBOV (isolate Mayinga [GenBank accession No. AF086833], or isolate Makona-Gueckedou-C07 [GenBank accession no. KJ660347]) were added to the serum dilutions. Following incubation for 1 hour at 37°C, Vero cell suspension was added. Plates were then incubated at 37°C with 5% CO_2_, and cytopathic effects were evaluated at 7 (Mayinga) or 9 (Makona-Gueckedou-C07) days after infection. Neutralization titers were calculated as the geometric mean titer (GMT) of 4 replicates.

### RNA Extraction and PCR Assay

Extraction of RNA was performed using the MagnaPure 96 small-volume RNA kit (Roche). Plates were loaded onto the MagnaPure 96 automated extraction robot, and RNA was eluted in 60 µL of nuclease free water. Target amplification was performed using primers to EBOV GP [19], using the Fast Virus qRT-PCR kit (Qiagen). Analysis was performed using the ABI 7500 (Applied Biosystems) at the following cycling conditions: 50°C for 10 minutes and 95°C for 30 seconds, followed by 40 cycles at 95°C for 15 seconds and 60°C for 3 seconds. Temperature cycling was set to maximum ramp speed, and data were acquired and analyzed using the ABi 7500 on-board software with a threshold set to 0.05. A standard curve of known quantities of an RNA transcript at was run in parallel on each PCR plate for assessing the number of genome copies per reaction.

### Animals

Female adult Dunkin-Hartley guinea pigs were used for animal infection studies, with weights of 250–350 g (Harlan Laboratories, United Kingdom). Animals were supplied with catheters inserted into the jugular vein, to allow intravenous access [20]. For procedures, guinea pigs were anaesthetized with 1.5%–2% isoflurane in oxygen until full sedation was achieved. Food and sterile water were available ad libitum. All procedures were undertaken according to the United Kingdom Animals (Scientific Procedures) Act 1986. Studies were conducted under Establishment Licence reference PEL PCD 70/1707 with Project Licence PPL 30/3247. A power calculation along with the Fisher exact test were performed using software G*Power, version 3.0.10, to determine group sizes for the experiments. A group size of 6 met a power of 0.8 and an α of 0.05.

EBOV stock was diluted in sterile PBS to prepare approximately 10^3^ TCID_50_ in a 0.2-mL volume and subcutaneously inoculated with the virus suspension in the lower right quadrant of the back. Virus was back titrated on VeroE6 cells (actual dose, 640 TCID_50_). EBOTAb was delivered intravenously starting at either 6, 48, or 72 hours following EBOV challenge. Repeat administrations were delivered, equating to 10, 8, and 7 doses for the 6-, 48-, and 72-hour groups, respectively. A volume of 0.5 mL containing 25 mg of ovine immunoglobulin was administered at each treatment.

Animals were weighed and temperatures recorded daily via an indwelling temperature chip. Clinical signs were monitored at least twice daily, and the following numerical score was assigned for analysis: 0 (normal), 2 (ruffled fur), 3 (lethargy, pinched, hunched, and wasp waisted); 5 (labored breathing, rapid breathing, and inactivity), and 10 (immobile). A set of humane clinical end points was defined to prevent unnecessary suffering (ie, 20% weight loss or 10% weight loss and a clinical symptom).

### Viral Load

Blood samples were collected in RNAprotect animal blood tubes (Qiagen), mixed, and stored at −80°C until processing. For processing, 200 µL of tissue homogenate or blood solution was transferred to 600 µL of RLT buffer (Qiagen) for RNA extraction and PCR analysis.

### Histologic Analysis

Samples of liver and spleen were placed in 10% neutral buffered formalin for at least 21 days and processed routinely in paraffin wax. Sections were cut into sections of 3–5 µm, stained with hematoxylin and eosin, and examined microscopically. For immunohistochemistry analysis, sections were stained for EBOV antigen, using the Leica BondMax (Leica Biosystems) and the Leica Bond Polymer Refine Detection kit (Leica Biosystems). An antigen-retrieval step was included for 10 minutes, using the Bond Enzyme Pretreatment kit, enzyme 3 (3 drops). A rabbit polyclonal anti-EBOV VP40 antibody (IBT Bioservices; dilution 1:2000) was incubated with the slides for 60 minutes. DAB chromogen and hematoxylin counterstains were used to visualize the slides.

## RESULTS

### Expression of Recombinant Glycoprotein

**Figure 1.**
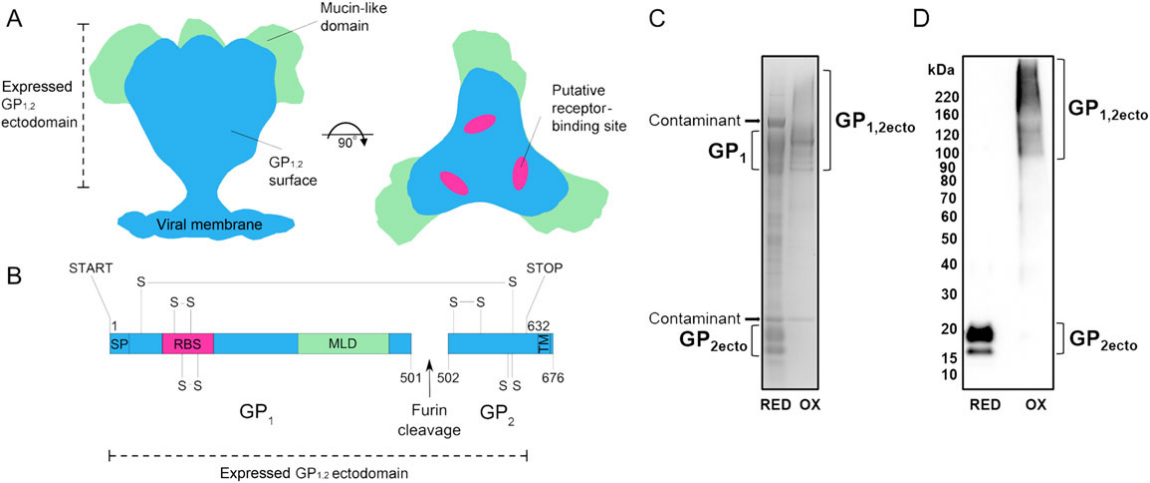
Organization, expression, and analysis of the Ebola virus glycoprotein (EBOV-GP). *A*, Schematic of the EBOV-GP_1,2_ trimer, based on cryo–electron microscopy (Electron Microscopy Data Bank accession nos. 6003 and 6004) [18]. The protein surface is shown in blue, the mucin-like domain in light green, and the putative receptor binding sites [21] in pink. *B*, Domain organization of EBOV-GP_1,2_. The transmembrane (TM) domain is followed by a short cytoplasmic tail (residues 672–676). The disulphide bridges (-S-S-) and the residue range present in the expressed EBOV GP_1,2ecto_ construct (START, STOP) are indicated. MLD, mucin-like domain; RBS, putative receptor binding site; SP, signal peptide. *C* and *D*, Coomassie-stained sodium dodecyl sulfate polyacrylamide gel electrophoresis (*C*) and Western blot analysis (*D*) of purified EBOV GP_1,2ecto_, run under reducing (RED) and nonreducing (OX) conditions, following a 1-step immobilized metal affinity chromatography (IMAC) purification. Protein contaminants in the sample, derived from either human embryonic kidney cell culture or fetal bovine serum, are annotated in panel *C*. Size exclusion analysis of the IMAC-purified sample is shown in Supplementary Figure 1. In panel *D*, EBOV GP_1,2ecto_ was detected with a primary antibody specific to the hexa-histidine tag encoded at the C-terminus of the construct.

EBOV-GP_1,2ecto_ was transiently expressed in HEK293 T cells and purified by IMAC (Figure 1*A* and 1*B* and Supplementary Figure 1). The resultant protein was produced at a level of approximately 2 mg per liter of cell supernatant. SDS-PAGE analysis and Western blot analysis revealed proper furin-dependent cleavage of the EBOV-GP_1,2ecto_ into GP_1_ and GP_2ecto_ subunits (Figure 1*C*).

### Binding of Ovine Immunoglobulin to EBOV GP

**Figure 2.**
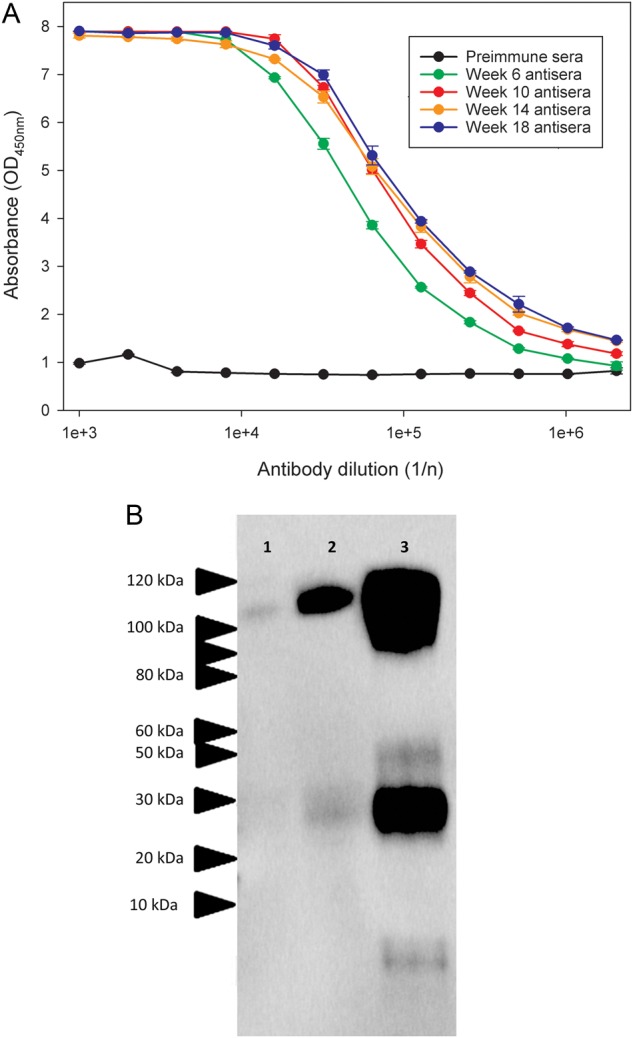
Binding of ovine sera after immunization to Ebola virus glycoprotein (EBOV-GP). *A*, Sheep were immunized with recombinant EBOV-GP_1,2ecto_ at 4 weekly intervals, and binding of antisera pools to the EBOV-GP_1,2ecto_ was assessed by an indirect enzyme-linked immunosorbent assay. *B*, Western blot analysis of purified ovine immunoglobulin G, showing recognition of protein isolates from VeroE6 cells infected with EBOV strains 1976/Yambuku-Ecran, 2014/Makona, and 2014/UKexSierraLeone in lanes 2, 3, and 4, respectively. Protein lysates for Western blot analysis were derived from VeroE6 cells infected with a multiplicity of infection (MOI) of 0.5 and harvested 72 hours (1976/Yambuku-Ecran and 2014/Makona) or 168 hours (2014/UKexSierraLeone) after infection. Estimated molecular masses are indicated.

Antisera pools from sheep immunized with recombinant EBOV-GP_1,2ecto_ exhibited strong antibody responses at week 6, which increased in blood specimens obtained on subsequent dates (Figure 2*A*). The 50% binding titers at weeks 6, 10, 14, and 18 were 1:52 000, 1:84 000, 1:95 000, and 1:120 000, respectively. Western blot analysis demonstrated antibody recognition of several EBOV strains: 1976/Yambuku-Ecran, 2014/Makona, and 2014/UKexSierraLeone (Figure 2*B*). Variations in binding were likely due to the different times after infection that proteins were prepared. EBOTAb was prepared from pools of antisera, and small-scale affinity chromatography revealed an average specificity of 10.2%.

### Inhibition of EBOV Infection by EBOTAb

EBOTAb was assessed for neutralization activity, with results showing a GMT of 11 585 and 9743 against the Mayinga and Makona EBOV strains, respectively. For both strains, the GMT for the control ovine IgG was <4.

### Survival and Clinical Observations

**Figure 3.**
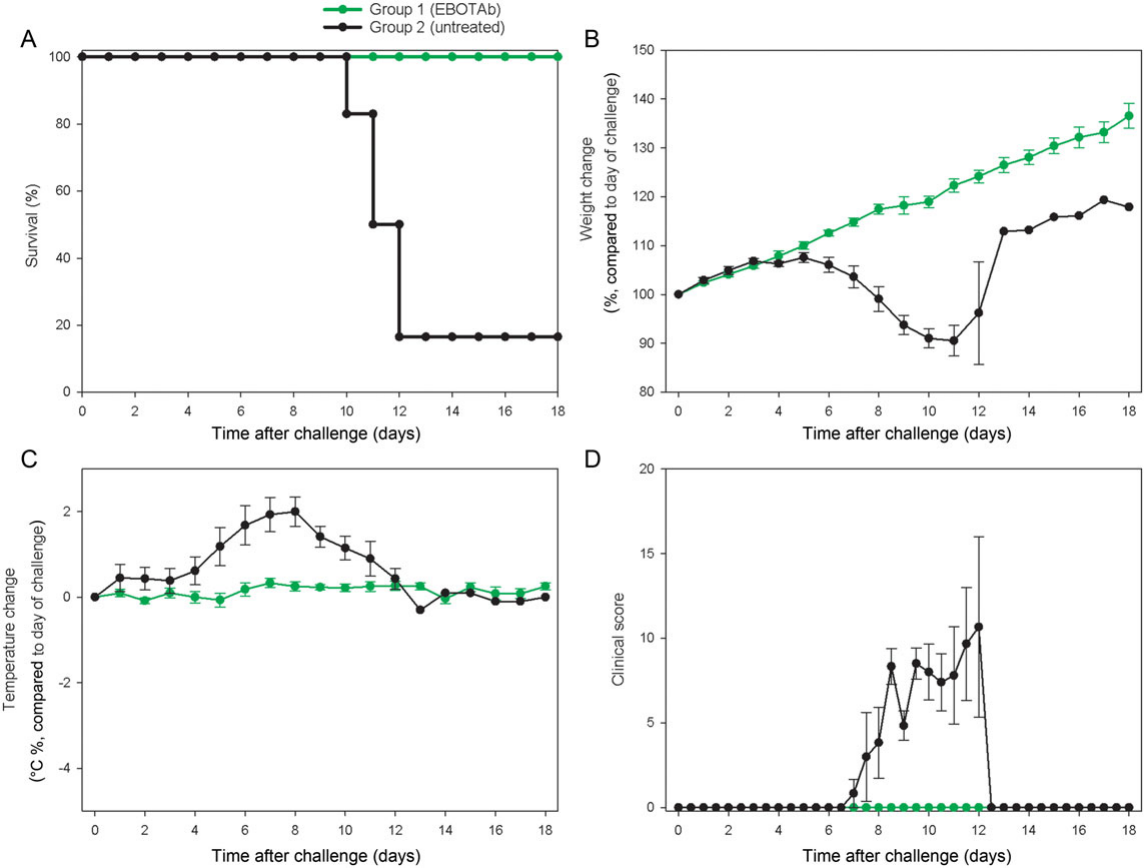
Survival and clinical overview of guinea pigs treated with EBOTAb 6 hours after challenge. Guinea pigs were challenged with Ebola virus (EBOV), and 25 mg of EBOTAb was administered intravenously in a volume of 0.5 mL 6 hours after challenge, followed by daily dosing until day 6 and dosing every 2 days until day 12 (group 1). Untreated animals received no compound (group 2). *A*, Survival analysis between the EBOTAb-treated group and untreated animals. *B*, Weight changes, showing percentage differences from values on the day of challenge. *C*, Temperature differences in animals compared to baseline taken on day of challenge. *D*, Clinical scores of animals after challenge. In panels *B–D*, mean results are shown for animals still surviving in all groups, with error bars denoting standard errors.

When first administered 6 hours after challenge, 100% survival was reached in 6 EBOTAb-treated animals, compared with 17% survival in untreated animals (1 of 6; Figure 3*A*), a statistically significant improvement (*P* = .005, by log-rank survival analysis). Animals treated with EBOTAb showed no evidence of weight loss after challenge (Figure 3*B*) and did not exhibit any increase in temperature (Figure 3*C*) or clinical signs (Figure 3*D*).

**Figure 4.**
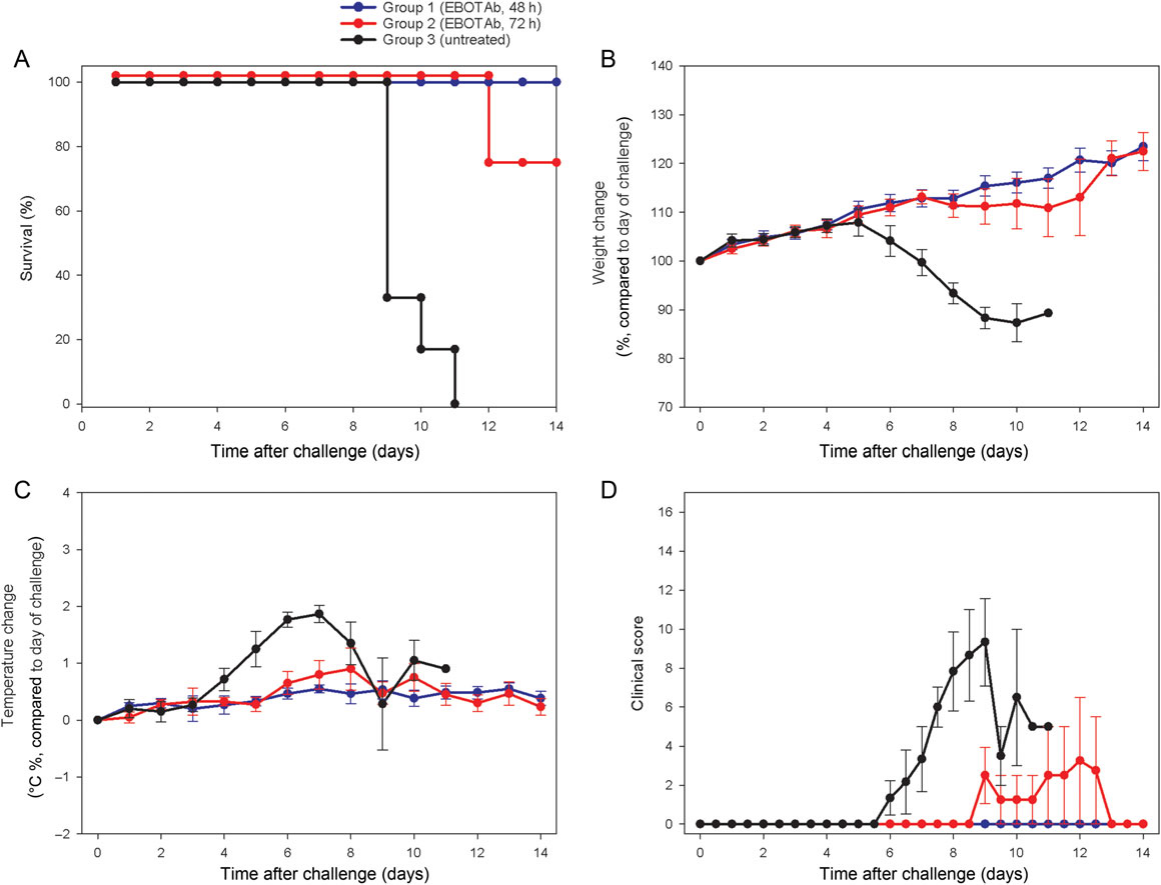
Survival and clinical overview of guinea pigs treated with EBOTAb 48 and 72 hours after challenge. Guinea pigs were challenged with Ebola virus (EBOV), and 25 mg of EBOTAb was administered intravenously in a volume of 0.5 mL 48 hours (group 1) or 72 hours (group 2) after challenge. EBOTAb was administered daily until day 7, followed by treatment every 2 days until day 11. Untreated animals (group 3) received no compound. *A*, Survival analysis between the EBOTAb-treated groups and untreated animals. *B*, Weight changes, showing percentage differences from values on the day of challenge. *C*, Temperature differences in animals, compared with baseline, on the day of challenge. *D*, Clinical scores of animals after challenge. In panels *B–D*, mean results are shown of animals still surviving in all groups, with error bars denoting standard errors.

A second experiment focused on extending the treatment window, with EBOTAb first administered either 48 hours or 72 hours after challenge. Results showed 100% (6 of 6 animals) and 75% (3 of 4 animals) survival in the 4-hour8 and 72-hour postchallenge groups, respectively (Figure 4*A*), a statistically significant result (untreated vs 48 hours, *P* < .001, by log-rank survival analysis; untreated vs 72 hours, *P* = .002, by log-rank survival analysis). Similarly, clinical parameters, including weight, temperature, and clinical signs were reduced in EBOTAb-treated animals as compared to controls (Figure 4*B*–*D*).

### Viral Load Analysis

**Figure 5.**
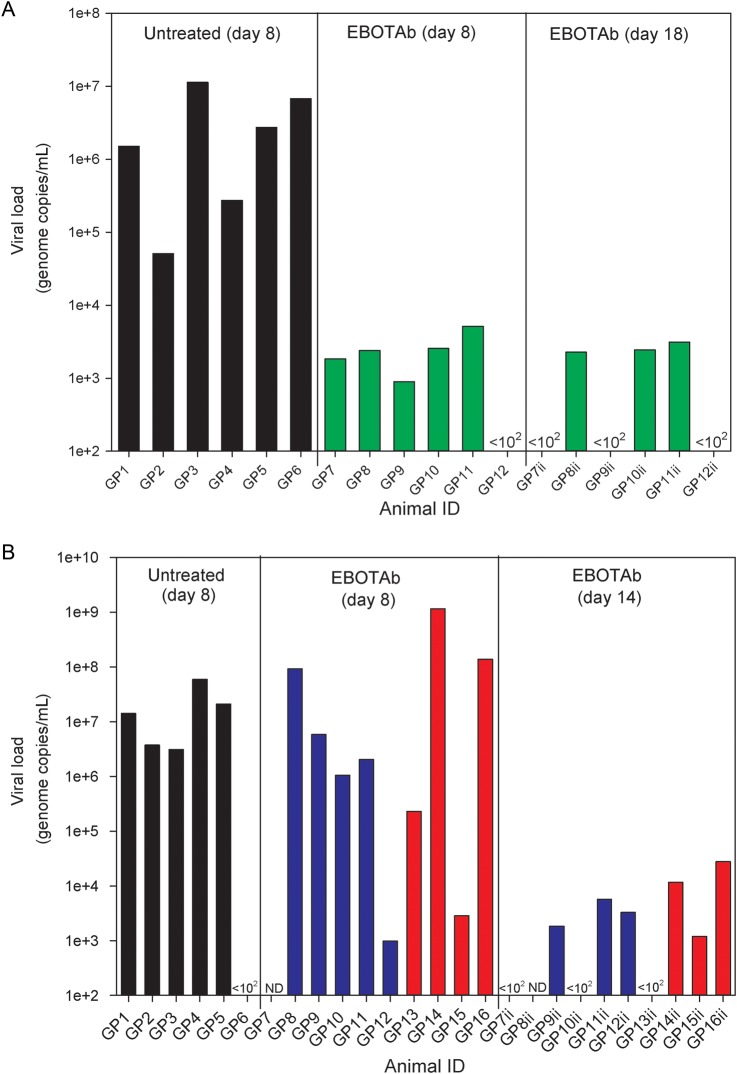
Viral genome copies in the blood and tissue of Ebola virus (EBOV)–challenged guinea pigs treated with EBOTAb and untreated controls. *A*, RNA levels in blood at day 8 after challenge and in EBOTAb-treated animals at day 18 in animals first treated with EBOTAb 6 hours after challenge (green bars). *B*, RNA levels in blood at day 8 after challenge and in animals that survived to day 14 among animals treated with EBOTAb starting 48 (blue bars) and 72 (red bars) hours after challenge. Abbreviations: GP, guinea pig; ND, not done.

Animals first treated 6 hours after challenge had a significant reduction in genome copies at day 8 (*P* = .005, by the Mann–Whitney test; Figure 5*A*). When administration of EBOTAb was delayed further, animals whose treatment was delayed until 48 hours and 72 hours after challenge had levels comparable to those in untreated animals at day 8 (*P* > .05, by the Mann–Whitney test; Figure 5*B*). At the experimental end points on day 18 or day 14 after challenge, viral levels in blood were similar in surviving animals from groups first treated 6, 48, or 72 hours after challenge.

### Histopathologic Analysis

In untreated control animals, prominent lesions suggesting infection with EBOV were observed in the spleen and liver. When EBOTAb was delivered 6 hours after challenge, lesions suggesting infection with EBOV were absent in the spleen and liver of all animals, and viral antigen was not detected (Table 1).

**Table 1.** Severity of Histological Lesions and Presence of Viral Antigen in Spleen and Liver of Ebola Virus–Challenged Animals After Treatment With EBOTAb 6 Hours After Challenge

Site, Lesion	Untreated Group						EBOTAb Group					
	GP1	GP2	GP3	GP4	GP5	GP6	GP7	GP8	GP9	GP10	GP11	GP12
Liver												
Hepatocyte vacuolation—macrovascular	WNL	Mod	WNL	Mkd	Mod	Mod	WNL	WNL	WNL	WNL	WNL	WNL
Focal, hepatocellular necrosis	Mild	Mild	Min	Mkd	Mod	Mild	WNL	WNL	WNL	WNL	WNL	WNL
Mineralization	WNL	Min	Min	Min	Mod	Mild	WNL	WNL	WNL	WNL	WNL	WNL
Spleen (red pulp)												
Congestion	Mild	Mod	Min	Mod	Mod	Mkd	WNL	WNL	WNL	WNL	Min	Mild
Scattered single cell degeneration/loss	Mod	Mod	WNL	Mild	Mod	Mod	WNL	WNL	WNL	WNL	WNL	WNL
Patchy PMN infiltration	Mod	Mod	WNL	Mild	Mod	Mod	WNL	WNL	WNL	WNL	WNL	WNL
Spleen (white pulp)												
Scattered single-cell necrosis	Mild	Mild	Min	Mod	Mild	Mod	WNL	WNL	WNL	WNL	WNL	WNL
Lymphocyte depletion	WNL	Mild	Min	Min	Mod	Mod	WNL	WNL	WNL	WNL	WNL	WNL
Presence of viral antigen												
Liver	Yes	Yes	No	Yes	Yes	Yes	No	No	No	No	No	No
Spleen	Yes	Yes	No	Yes	Yes	Yes	No	No	No	No	No	No

Abbreviations: GP, guinea pig; Min, minimal; Mkd, marked; Mod, moderate; PMN, polymorphonuclear leukocytes; WNL, within normal limits.

When EBOTAb treatment was delayed to 48 and 72 hours after challenge, lesions and the presence of viral antigen were noted in the spleen and liver of all untreated animals, confirming infection with EBOV (Table 2). Lesions consistent with EBOV infection were not present in the animals treated with EBOTAb 48 hours after challenge. In the 4 animals treated with EBOTAb 72 hours after challenge, lesions consistent with EBOV infection were observed, and viral antigen was detected in the spleen of 2 animals and the liver of all animals.

**Table 2.** Severity of Histological Lesions and Presence of Viral Antigen in Spleen and Liver of Ebola Virus–Challenged Animals After Treatment With EBOTAb 48 and 72 Hours After Challenge

Site, Lesion	Untreated Group						EBOTAb Group, Time After Challenge									
							48 h						72 h			
	GP1	GP2	GP3	GP4	GP5	GP6	GP7	GP8	GP9	GP10	GP11	GP12	GP13	GP14	GP15	GP16
Liver																
Hepatocyte vacuolation—macrovesicular^a^	Mod	Mod	Mod	Mod	Mild	Min	WNL	WNL	WNL	WNL	WNL	WNL	WNL	Mod	WNL	WNL
Hepatocyte vacuolation—microvesicular^b^	WNL	WNL	WNL	WNL	WNL	WNL	WNL	WNL	Mild	Min	Mod	Mod	WNL	WNL	WNL	WNL
Focal necrosis	Mod	Mod	Mod	Mod	Mod	Mod	WNL	WNL	WNL	WNL	WNL	WNL	Min	Mkd	WNL	Mod
Focal mineralization	Mod	Mod	Mod	Mod	Mod	Mod	WNL	WNL	WNL	WNL	WNL	WNL	Min	Mkd	WNL	Mod
Focus of hepatocyte rarefaction^c^	WNL	WNL	WNL	WNL	WNL	WNL	WNL	Min	WNL	WNL	WNL	WNL	WNL	WNL	Mild	WNL
Foci of lymphocytes^d^	WNL	WNL	WNL	WNL	WNL	WNL	Min	Mild	Min	Min	Min	Min	Min	WNL	Mild	Mild
Spleen (red pulp)																
Congestion	Mkd	Mkd	Mild	Mkd	Mod	Mild	Mild	Mild	Min	Min	Mild	Mild	Min	Mkd	Min	Min
Scattered single-cell degeneration/loss	Mod	Mod	Min	Mild	Mod	Mod	WNL	WNL	WNL	Min	Min	WNL	WNL	Mkd	WNL	Mod
Patchy polymorph infiltration	Mod	Mod	Mild	Mod	Min	Mild	Min	Min	WNL	Min	Min	WNL	WNL	Mod	WNL	WNL
Spleen (white pulp)																
Scattered single-cell necrosis	Mod	Mod	Mod	Mod	Mod	Mod	WNL	WNL	WNL	Min	WNL	WNL	WNL	Mild	WNL	WNL
Lymphocyte depletion	Mild	Mild	Mild	Mild	Mkd	Mod	WNL	WNL	WNL	WNL	WNL	WNL	WNL	Mild	WNL	WNL
Presence of viral antigen																
Liver	Yes	Yes	Yes	Yes	Yes	Yes	No	No	No	Yes	No	No	No	Yes	No	Yes
Spleen	Yes	Yes	Yes	Yes	Yes	Yes	No	No	No	No	No	Yes	Yes	Yes	No	Yes

Abbreviations: GP, guinea pig; Min, minimal; Mkd, marked; Mod, moderate; WNL, within normal limits.

^a^ Consistent with fat.

^b^ Consistent with glycogen.

^c^ Including pale-staining cytoplasm and scattered inflammatory cells.

^d^ Located in parenchyma and/or periportal areas.

## DISCUSSION

In the present study, the EBOV-GP_1,2ecto_ used to raise the ovine antisera was proven to be an excellent immunogen, with high levels of pAb present binding with high avidity. The procedure used to manufacture EBOTAb from pools of the antisera has been used for several years to provide an antivenom that is used widely in West Africa [22]. Ovine immunoglobulin preparations also confer additional benefits, such as stability at ambient temperatures and tolerance to tropical temperatures without loss in binding activity [23].

The binding of EBOTAb to GP_1_, GP_2_, and precursor soluble GP (sGP) observed in the Western blot is encouraging and suggests that the compound will exert multiple effects. GP_1_ is responsible for binding cellular receptors, whereas GP_2_ is a fusion protein that facilitates merger of the host and viral membranes. In contrast, large quantities of sGP are secreted into the extracellular fluid compartment in large amounts and, because it shares many epitopes with GP_1,2_, may be contribute to viral virulence and immune evasion via a process called “antigenic subversion” [24]. sGP may also bind Toll-like receptor 4 (TLR4) on dendritic cells and macrophages, triggering the release of cytokines and other factors that contribute to vascular leakage, inflammation, and ultimately poor clinical outcome [25].

Use of a neutralization assay currently used in clinical vaccine trials [26] revealed that EBOTAb had strong neutralization activity against 2 variants of EBOV: Mayinga, isolated from the original 1976 outbreak [27], and Makona-Gueckedou-C07, recovered during the ongoing outbreak affecting West Africa [3]. The currently circulating species of EBOV (Makona) has likely resulted from the natural variation of an ancestral EBOV. Given the opportunity for variation, it is conceivable that a new species of *Ebolavirus* may emerge and pose a threat to human health, as has been observed in the genus already. Many other examples exist in virology, and recent reports of African henipavirus spillover into human populations [28] are equally significant. Clearly, it will be beneficial if EBOTAb proves to be equally effective against such new strains. A polyclonal preparation should recognize a breadth of epitopes against the EBOV GP, including so-called areas of vulnerability [10]. It is feasible that mAb-based products may be compromised by genomic drift [29]. Whereas recent structural modeling studies have demonstrated that none of the accrued mutations in the EBOV strains from Guinea and Sierra Leone fall directly within the epitopes of the ZMapp antibodies [10], such mutants are possible, as evidenced by repeat passage of a pseudotyped virus (vesicular stomatitis virus expressing EBOV GP) in the presence of ZMAb antibodies [30].

Currently, there are no firm data relating to the optimum schedule for administrating exogenous antibodies. Many parameters are likely to influence such a schedule, especially the number of viruses to be neutralized. The latter will reflect the initial infectious dose and the time that elapses between the primary exposure and the start of immunotherapy. Additionally, viruses within the cells are beyond the reach of antibodies, and the subsequent treatment schedule must be cognizant of the viral replication time and replication number. The EBOTAb regimen should be refined to further define its efficacy and optimize potential levels for clinical use.

The initial guinea pig study in the present work examined the use of EBOTAb in a short duration (6 hours) after exposure to EBOV and showed 100% protection. This raises the potential of prophylactic use of antibodies, since this can be initiated rapidly in members of the public who have had intimate contact with a patient or corpse of a victim of EBOV disease. It may also be considered for physicians, nurses, and laboratory staff who have been exposed as a result of contact with a patient or their bodily secretions or contaminated samples [31]. It may be appropriate to combine such prophylactic use of exogenous antibodies with active immunization using a suitable vaccine, as this is currently provided routinely after possible exposure to rabies [32], or with complementary therapeutic treatments, such as type I interferon [33]. Survival of a single animal in the untreated group was likely due to the use of outbred animals, as individual genetic variations can affect disease progression.

Treatment can be defined as any means to improve the chances of survival of a patient in whom a diagnosis of EBOV infection has been made clinically and confirmed by a suitable laboratory diagnostic procedure. Based on studies undertaken in nonhuman primates, this will not be until at least the third day after exposure [33]. This was a further rationale for a second series of guinea pig studies, in which EBOTAb was not started until either 48 hours or 72 hours after challenge, using the same dose but delivered on 8 or 7 occasions, respectively, as opposed to 10 occasions, thereby reducing the total amount of IgG given per course from 250 mg to 175–200 mg. All guinea pigs in the 48-hour postchallenge group survived and experienced no pyrexia or retardation in their rate of weight gain or other clinical manifestations. However, when treatment was delayed for 72 hours, one of the 4 guinea pigs developed a fever, lost weight, and died. This waning of efficacy 3 days after challenge has also been observed in guinea pigs treated with combinations of monoclonal antibodies [33]. It should be noted that our studies lasted for up to 18 days after challenge and that viral RNA was still present in some the samples tested at this time point, although no antigen staining was observed with immunohistochemical staining. It is possible that the PCR assay detected RNA fragments from immune cells. Further studies would be required to extend the observation window after challenge and determine whether the presence of viral RNA equates to viable EBOV.

To further refine EBOTAb, large-scale affinity chromatography may be used to separate specific pAb from the remaining IgG, thereby reducing the amounts needed for therapeutic doses by several fold. This is used in the manufacture of one of MicroPharm's antivenoms, ViperaTAb, which is used to treat snake envenomation in the United Kingdom and Scandinavia [34, 35]. However, such a step adds considerably to the cost, and a primary aim of the present research is to provide a product affordable for those who will most need it. As our results demonstrate, within a 6-month period, a promising therapy against EBOV has been developed and has successfully been shown to protect guinea pigs against EBOV infection up to 3 days after exposure. EBOTAb is cost-effective, thus reducing the economic burden that results from the emergence of highly pathogenic viruses, especially in developing regions.

## Supplementary Data

Supplementary materials are available at http://jid.oxfordjournals.org. Consisting of data provided by the author to benefit the reader, the posted materials are not copyedited and are the sole responsibility of the author, so questions or comments should be addressed to the author.

Supplementary Data
