# Supplementary material for: Development of a Cost-effective Ovine Polyclonal Antibody-Based Product, EBOTAb, to Treat Ebola Virus Infection
Source: J Infect Dis. 2015 Dec 28;213(7):1124–33. doi: 10.1093/infdis/jiv565 (PMC4779302; doi:10.1093/infdis/jiv565)
Supplement: Supplementary Data [file supp_213_7_1124__index.html]

Supplementary Data 

# Development of a Cost-effective Ovine Polyclonal Antibody-Based Product, EBOTAb, to Treat Ebola Virus Infection

## Supplementary Data

Supplementary Data

- Supplementary Figure 1 - tif file
