# Supplementary figures and images for: Development of a Cost-effective Ovine Polyclonal Antibody-Based Product, EBOTAb, to Treat Ebola Virus Infection
Source: J Infect Dis. 2015 Dec 28;213(7):1124–33. doi: 10.1093/infdis/jiv565 (PMC4779302; doi:10.1093/infdis/jiv565)

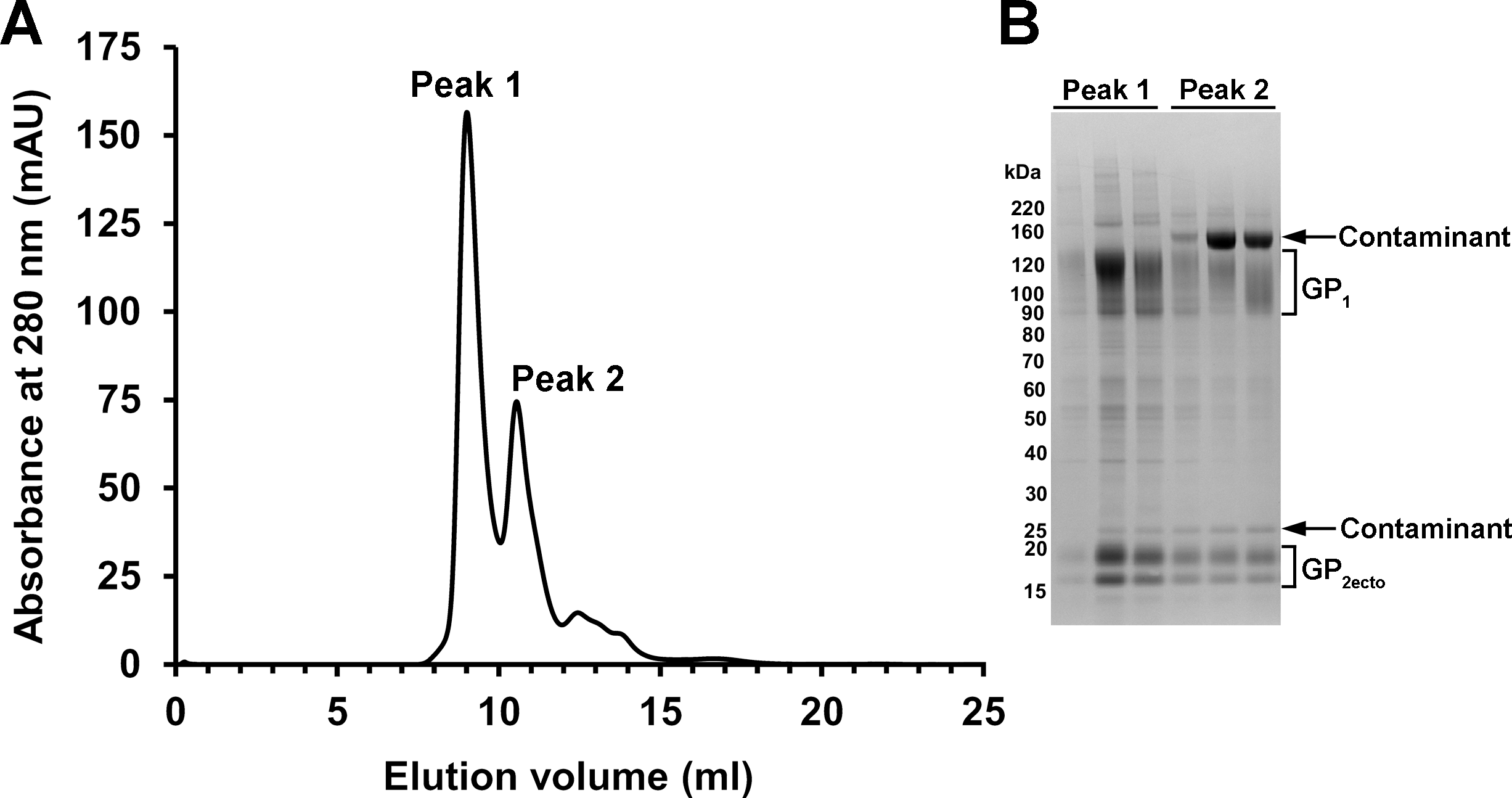

Supplement: Supplementary Data [file supp_jiv565_jiv565supp_fig1.tif]
